# DeepMPF: deep learning framework for predicting drug–target interactions based on multi-modal representation with meta-path semantic analysis

**DOI:** 10.1186/s12967-023-03876-3

**Published:** 2023-01-25

**Authors:** Zhong-Hao Ren, Zhu-Hong You, Quan Zou, Chang-Qing Yu, Yan-Fang Ma, Yong-Jian Guan, Hai-Ru You, Xin-Fei Wang, Jie Pan

**Affiliations:** 1 School of Information Engineering, Xijing University, Xi’an, 710100 China; 2 School of Computer Science, Northwestern Polytechnical University, Xi’an, 710129 China; 3 Institute of Fundamental and Frontier Sciences, University of Electronic Science and Technology of China, Chengdu, 610054 China; 4 Department of Galactophore, The Third People’s Hospital of Gansu Province, Lanzhou, 730020 China

**Keywords:** Drug–protein interactions, Multi-modal, Meta-path, Sequence analysis, Joint learning, Natural language processing

## Abstract

**Background:**

Drug-target interaction (DTI) prediction has become a crucial prerequisite in drug design and drug discovery. However, the traditional biological experiment is time-consuming and expensive, as there are abundant complex interactions present in the large size of genomic and chemical spaces. For alleviating this phenomenon, plenty of computational methods are conducted to effectively complement biological experiments and narrow the search spaces into a preferred candidate domain. Whereas, most of the previous approaches cannot fully consider association behavior semantic information based on several schemas to represent complex the structure of heterogeneous biological networks. Additionally, the prediction of DTI based on single modalities cannot satisfy the demand for prediction accuracy.

**Methods:**

We propose a multi-modal representation framework of ‘DeepMPF’ based on meta-path semantic analysis, which effectively utilizes heterogeneous information to predict DTI. Specifically, we first construct protein–drug-disease heterogeneous networks composed of three entities. Then the feature information is obtained under three views, containing sequence modality, heterogeneous structure modality and similarity modality. We proposed six representative schemas of meta-path to preserve the high-order nonlinear structure and catch hidden structural information of the heterogeneous network. Finally, DeepMPF generates highly representative comprehensive feature descriptors and calculates the probability of interaction through joint learning.

**Results:**

To evaluate the predictive performance of DeepMPF, comparison experiments are conducted on four gold datasets. Our method can obtain competitive performance in all datasets. We also explore the influence of the different feature embedding dimensions, learning strategies and classification methods. Meaningfully, the drug repositioning experiments on COVID-19 and HIV demonstrate DeepMPF can be applied to solve problems in reality and help drug discovery. The further analysis of molecular docking experiments enhances the credibility of the drug candidates predicted by DeepMPF.

**Conclusions:**

All the results demonstrate the effectively predictive capability of DeepMPF for drug-target interactions. It can be utilized as a useful tool to prescreen the most potential drug candidates for the protein. The web server of the DeepMPF predictor is freely available at http://120.77.11.78/DeepMPF/, which can help relevant researchers to further study.

**Supplementary Information:**

The online version contains supplementary material available at 10.1186/s12967-023-03876-3.

## Introduction

In the post-genomic era, the prediction of drug-target interaction (DTI) plays a pivotal role in drug discovery and drug repositioning, which is dedicated to exploring new therapeutic use for existing drugs by narrowing down the search scope of drug candidates to improve the efficiency of drug development [1]. According to the statistics, 13–15 years need to be taken to approve a new drug, and the development cost ranges from US $200 million to US $3 billion [2]. Since the concept of polypharmacology [3] emerged, researchers can understand drug side effects and find their new usage, namely drug repositioning, which can save money and time in developing a new treatment, [4]. For example, *Imatinib Mesylate* was only thought to treat *Leukemia* through interacting with *Bcr-Abl* fusion gene. Later, *Imatinib Mesylate* was found to cure *gastrointestinal stromal tumors* by interacting with *PDGF* and *KIT* [5, 6]. The finding processes of *thalidomide*, *bupropion* and *fluoxetine* also share similarities [7].

In previous work, drug repositioning and drug-target prediction have often been considered separately. In reality, these two tasks have intrinsic correlations due to the same drug feature space. Drugs indirectly alter biological pathways for treating diseases through modulating target activities, which can inextricably link the disease domain to the target domain [8, 9]. Therein, rapidly determining whether generating the interaction between a drug and a protein is a crucial key in accelerating the process of drug repositioning, which is important for understanding the mechanism of drug reaction [10, 11]. However, many human and financial resources have been consumed by traditional biological experiments [12]. For workload reduction of the wet-lab experiment, proposing the computational model to predict unknown DTI with considering the disease domain is urgently needed.

Well-accepted traditional calculation approaches for determining DTIs are grouped into two categories, ligand-based approach and molecular-docking-based approach [13, 14]. The first approach predicts interactions utilizing the similarity between the ligands of protein, which is limited by the information of known ligands per protein. The other approach utilizes the 3D structure of proteins to identify DTIs. However, if the 3D structure is unavailable, like membrane proteins, interaction identification will be a challenging task. With biological technology and high-throughput technology rapidly developing, several multi-omics data have been generated to provide diverse biological sources for drug-target prediction and drug repositioning [15–21]. Meanwhile, the enhanced performance of the computer promotes the chemogenomic computation approaches. Currently, the prediction of DTI can be regarded as a binary classification problem [22–25]. The chemogenomic methods can be divided into similarity-based methods and network-based methods, which extract and encode the information about drugs and targets into representation features to train predicting models [27, 28].

The similarity-based methods are based on the underly idea that similar drugs may share similar proteins, and vice versa. Shi et al. provided LRF-DTIs which exploits pseudo-position specific scoring matrix (PsePSSM) and FP2 molecular fingerprint to obtain the raw features, and after dimension reduction by Lasso, the random forest is used to classify [29]. Similar to LRF-DTIs, Pan et al. put forward a method innovatively using image processing algorithms of dual-tree complex wavelet transform (DTCWT) to extract evolutionary information of proteins and using molecular fingerprints to present drug information. Finally, rotation forest is utilized to classify [30]. However, due to these methods classifying through traditional machine learning models and single perspective information, the performance is limited and may miss some crucial feature information in the process of predicting. Many deep learning methods are proposed to solve the problems [27]. Wen et al. proposed the model of DeepDTIs to identify unknown DTIs, which automatically extracted structure and sequence information, and predicted by the deep belief network (DBN) [31]. Huang et al. designed an augment transformer encoder to capture the semantic relation of substructure and spliced features of drug and protein to put them into the deep neural network (DNN) for prediction [32]. Chen et al. introduced NeurTN to identify DTIs, which made full use of the information on drugs, targets and diseases through tensor algebra [33]. A model of DeepCDA is designed to test binding affinity by Abbasi et al., which learns local substructure patterns through convolution layers and LSTM layers to enhance the features, and trains the feature encoder network [34]. The methods mentioned above are mainly based on exploring advanced and reasonable feature-extracting approaches to capture the information of drugs and proteins, which can be utilized to classify through the way of traditional machine learning or deep learning. The biggest advantage of similarity-based methods is they can predict new drugs and new proteins. Fully using biological characteristics can bring strong scalability and generalization ability to the model. However, these methods cannot capture deep interactions between drugs and proteins, and due to single-sided biological structure information only being considered, if missing some information about drugs or proteins, these models will not work.

The network-based method is based on an assumption that the drugs tend to interact with similar targets, and vice versa. The matrix factorization methods are usually proposed with the optimized regularization or profile kernel to predict DTIs [35, 36]. Recently, due to increasing multi-source data appearing, utilizing multiple types of biological functional objects as feature information has been getting lots of attention in academia. For example, Peng et al. and Shao et al. predicted DTIs by integrating various node information through Graph Convolution Network (GCN) [37, 40]. Similarly, Wan et al. proposed NeoDTI to predict DTIs, based on GCN integrating multi-type neighborhood information to advanced features through the neural network [39]. These methods of features diffusing according to the network structure ignore the direct association behavior semantic information of the network structure. To make better use of multiple features, Chen et al*.* drew a self-supervised framework to capture the node information of local and global perspectives from the heterogeneous network [38] and Soh et al. simply spliced the information of sequence and related biological entities into the long feature vector to enhance DTIs predicting [41]. These methods considered multi-type information. However, the way of integrating is too simple to obtain superior performance. Additionally, the rich semantic information of the meta-path of various schemas is ignored, which is crucial for analyzing heterogeneous networks and further improving the accuracy of DTI prediction [28]. Fu et al*.* developed 51 paths and multiplied each interaction matrix to generate representation according to the current path. Finally, the representation was used to predict DTIs by random forest [42]. Li et al. used a two-level neural attention mechanism to obtain latent features, which are mapped to the best projection space to generate scores by inner product [43]. Although, these methods fully used interactive semantics from link relationships and topological structure of different biomedical information networks, they are still single-modal methods. Hence, for avoiding the disadvantages of the similarity-based and network-based methods, there is a requirement to explore a computational method based on multi-modal, which simultaneously considers and effectively exploits features from multiple perspectives of protein–drug-disease association structure information, drug information, protein information and similarity information.

In this study, a novel deep learning framework DeepMPF is proposed, which is based on multi-modal representation learning, containing sequence modality, heterogeneous structure modality and similarity modality. As previous work mentioned, merged multiple information provides better generalization than any single information [85]. To make DeepMPF can be better applied in drug repositioning, the disease domain is fully considered in our model. Specifically, we first integrate protein–drug-disease association information to construct a biological heterogeneous network. For capturing heterogeneous structure information, six schemes of meta-paths are proposed to generate association behavior semantic sequences, which are exploited to fully learn node embedding vector through maximizing the probability of each center word. Second, considering the different biochemical properties, the sequence information of the drug and protein is extracted by the natural language processing (NLP) method and *3*-mers sparse matrix, respectively. Third, the similarity of structure also provides another important perspective. We respectively utilized Smith-Waterman scores [44] and SIMCOMP [45] to calculate the similarity for each pair of protein and drug. Finally, advanced features are generated through joint learning. We adopt binary-cross-entropy loss and backpropagation to train the model. The optimizer of adam is utilized to automatically adjust the learning rate. The results of five-fold cross-validation and comparison with state-of-the-art methods can demonstrate that DeepMPF is suitable for predicting DTI. Code is available at https://github.com/MrPhil/DeepMPF.

Recently, the COVID-19 pandemic is ongoing. To make our model of more practical significance, case studies of predicting DTI were conducted, which can be regarded as an application of drug repositioning, containing the targets related to COVID-19 treatment. Furthermore, the protein CYP3A4 related to HIV infection is utilized for research. In conclusion, our work indicated that DeepMPF can be utilized as the prescreening tool for predicting DTI in the molecular polypharmacological space. More meaningfully, we provide a computational platform for related researchers and biologists to prescreen the potential DTIs and further validate them through wet experiments.

## Materials and methodology

### Biological heterogeneous network

The gold standard dataset of DTIs worked by Yamanishi et al. [46] is widely utilized as a benchmark dataset. According to the type of protein, the dataset can be divided into four main datasets containing enzymes, G-protein-coupled receptors (GPCR), ion channels and nuclear receptors, which have been collected from DrugBank [16], BRENDA [47], KEGG ERITE [48] and Super-Target [49]. Due to the complete picture of association discovery among drug, target and disease being of significant importance to understanding the underlying molecular mechanisms [50], we collect and add the drug-drug interactions (DDIs) network and drug-disease associations (DDAs) network to the DTIs network for constructing the biological heterogeneous network. We first downloaded drug-related information from the database of DrugBank and disease-related information from the database of CTD [20], and then, four different heterogeneous networks are respectively constructed according to the four main datasets mentioned above to perform the subsequence experiments.

In the process of model learning, we employ the known DTIs as the positive samples and the rest of the drug-target pairs are seen as negative samples. Due to the severely imbalanced samples, we randomly choose the negative samples with the same number of positive samples to correct the bias. The positive and negative samples are split into train, validation and test sets with the proportion of 7:1:2. For applying DeepMPF to the drug repositioning task, we construct the dataset proDB to conduct case studies. In the proDB, the data of DTIs contains new DTIs downloaded from DrugBank (version 5.1.8) and the DTIs collected by Shi et al. [29], and the edge information of DDIs and DDAs is added similarly as described previously. Table 1 illustrates details of the number of various entities and interactions/associations in the five heterogeneous networks.

**Table 1:** The details of the DTIs gold standard datasets added other heterogeneous information

Data set	Interaction types	# of entity A	# of entity B	# of edge A-B
Enzymes	Protein–drug Interaction	662	445	2923
	Drug-Drug Interaction	248	248	6598
	Drug-Disease Association	356	3174	80,943
GPCRs	Protein–drug Interaction	95	223	635
	Drug-Drug Interaction	133	133	2775
	Drug-Disease Association	188	5257	80,077
Ion channels	Protein–drug Interaction	204	210	1476
	Drug-Drug Interaction	115	115	2875
	Drug-Disease Association	183	4438	65,951
Nuclear receptors	Protein–drug Interaction	26	54	90
	Drug–drug Interaction	43	43	254
	Drug-Disease Association	49	5509	50,032
proDB	Protein–drug Interaction	3004	3945	20,808
	Drug-Drug Interaction	1626	1626	194,264
	Drug-Disease Association	2485	7085	2,028,072

### Sequence information and similarity information

As two other perspectives of features for the multi-modal model, sequence and similarity information can ensure high generalization and strong stability. Additionally, rationally exploiting them not only can further improve the performance but also can provide the capability of identifying drugs outside the heterogeneous network. The sequence information of the drug and protein are respectively downloaded from DrugBank [16] and KEGG [15] databases. Benefiting from the work of Yamanishi et al. [46], we can directly utilize the similarity information from available data.

### Overview of methods

**Fig. 1:**
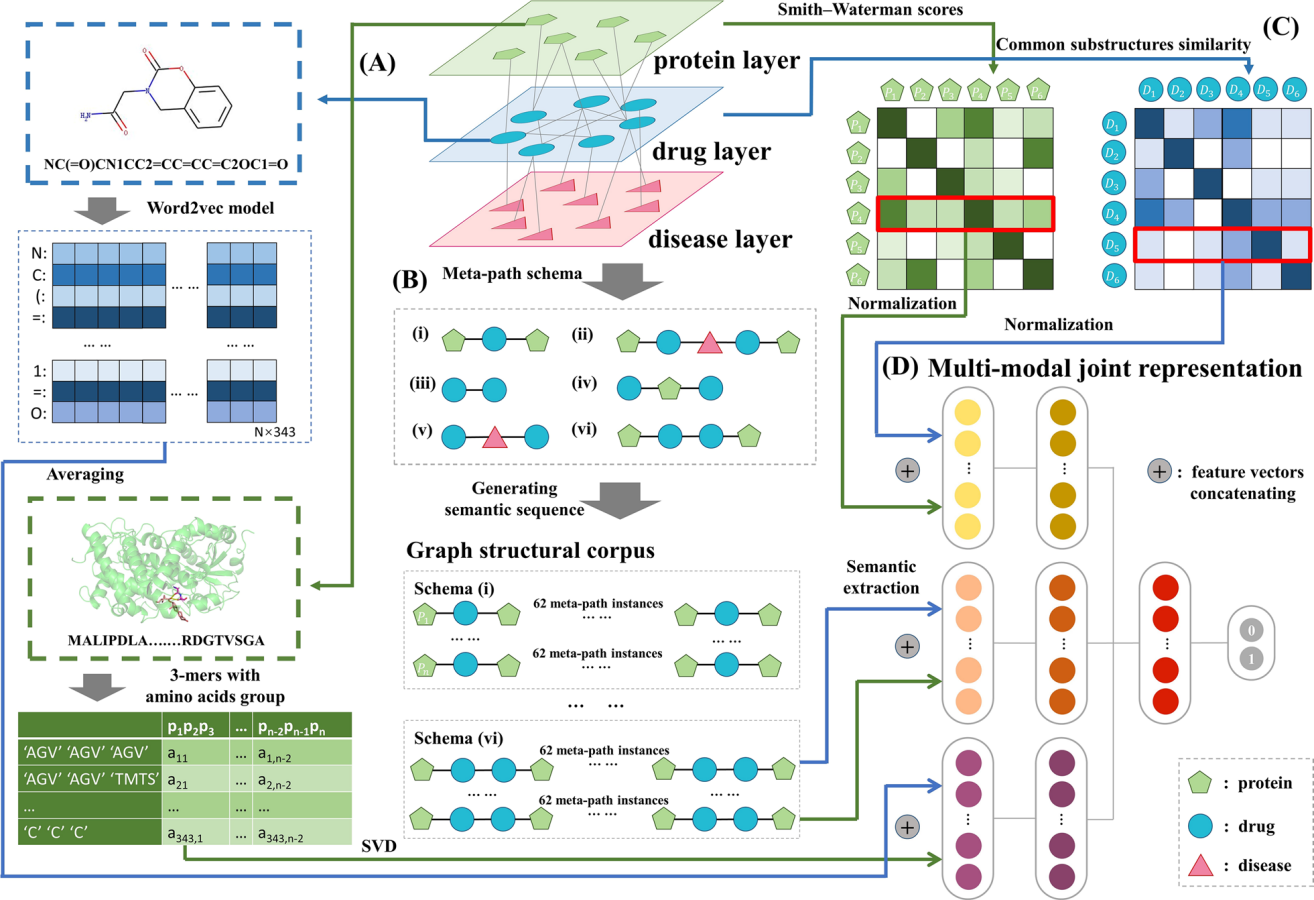
The architecture for the DeepMPF. The **A** and **C** respectively descript the extracting process of sequence and similarity information of each drug and protein in the heterogeneous network. The feature-extracting process **B** is under the heterogeneous structure information perspective

DeepMPF is a deep learning framework proposed to predict unknown DTIs based on multi-modal representation learning. When learning and understanding the same phenomenon, such as DTI, multi-modal representation learning can perform more robust identification by capturing invisible complementary information in individual modalities. Figure 1 shows the flowchart of the proposed framework. Our framework contains three single modalities which respectively represent sequence information perspective modality, heterogeneous structure information perspective modality and similarity information perspective modality. Then, to enhance feature representation, the multiple hidden layers fully fuse multiple information of different perspectives as advanced features, which are utilized to train a model. Finally, the features from test samples are fed into the trained modal to evaluate predictive performance. Next, we will elaborate on the whole flow of DeepMPF.

### Representation within sequence information perspective

Due to the difference in biochemical properties between the drugs and proteins, we employ different methods to extract effective features. For drug sequence information, we utilize the NLP method to learn, which benefits from the development of deep learning technology. Specifically, with representing the SMILES as drug sequences, each sequence of the drug is regarded as a sentence, which is used to construct a biochemical corpus, and each symbol in the sequence is seen as a word. Then, similar to the previous work [51], we calculate the embedding vectors of biochemical symbols through distributed representation vector learning method of word2vec [52, 53]. The model of CBOW calculates the probability of the appearance of the center word on the basis of the context word. At first, each word (symbol) is represented as one-hot vector *x*_*i*_ with *V*-dimension, where *V* means the number of words in the biochemical corpus. Given a length of sliding windows *c*, the center word can be denoted as the arithmetic average of the context word under the windows, as follows:

1 $$ h = \frac{1}{c}\omega^{{\text{T}}} (x_{1} + x_{2} + ... + x_{c} ) $$

where $\omega$ represents a learning weight matrix of the hidden layer. Then, through optimizing learning weight $\omega ^{\prime}$, approximating the occurrence probability of the actual center word *x*_*j*_ to 1 by the function:

2 $$E =  - \log P({x_j}|{x_1},{x_2},...,{x_c}) = \log \sum\limits_{i = 1}^V {\exp (\omega_i^{\prime T} \times h) - \omega_j^{\prime T}}  \times h,j \in [1,V]$$

where $\omega ^{\prime}_{i}$ indicates *j*-th row of the weight matrix $\omega ^{\prime}$. After embedding to drug semantic space, each sentence (SMILES of drug) can be represented as a matrix, whose rows mean symbol embedding vectors. To reduce dimension, the representation vector of drugs can be obtained through averaging by row. In this work, we set the length of sliding windows as 5 and set the embedding dimension as 343, which is the same as the embedding dimension of protein.

For protein sequence, to fully extract amino acid constituents and order information, the features of group-level amino acids are caught by the *3*-mers sparse matrix. In detail, according to the dipole moments and side-chain volume, the 20 amino acids are separated into 7 classes [54, 55], ‘AGV’, ‘ILFP’, ‘TMTS’, ‘HNQW’, ‘DE’, ‘RK’ and ‘C’, whose names are utilized to replace symbols of amino acids in the protein sequence. Afterward, based on *k*-mers, each protein of length *n* is represented as a sparse matrix *L*_*p*_, whose dimension is 7^* k*^ × *n*-(*k*-1) [56]. *L*_*p*_ is defined as follows:

3 $$ L_{p} = (e_{ij} ),i \in [0,7^{k} - 1],j \in [0,(n - (k - 1))] $$

4 $$ e_{ij} = \left\{ \begin{gathered} 1,{\text{ if }}p_{j} p_{j + 1} p_{j + 2} { = }k{ - }mer(i) \hfill \\ 0,{\text{ else}} \hfill \\ \end{gathered} \right. $$

The value of *k* is set to 3 which is regarded as an empirical parameter [57, 58]. And the feature of the conjoint triad *p*_*j*_*p*_*j*+*1*_*p*_*j*+*2*_ for each protein is shown in Table 2. Furthermore, the vector with the dimension of 343 can be obtained from *L*_*p*_ through the SVD method.

**Table 2:** *3*-mer sparse matrix of the protein sequence

	*p*_*1*_*p*_*2*_*p*_*3*_	*p*_*2*_*p*_*3*_*p*_*4*_	*…*	*p*_*n-2*_*p*_*n-1*_*p*_*n*_
‘AGV’ ‘AGV’ ‘AGV’	*e*_*11*_	*e*_*12*_	*…*	*e*_*1,n-2*_
‘AGV’ ‘AGV’ ‘ILFP’	*e*_*21*_	*e*_*22*_	*…*	*e*_*2,n-2*_
‘AGV’ ‘TMTS’ ‘AGV’	*e*_*31*_	*e*_*32*_	*…*	*e*_*3,n-2*_
…	*…*	*…*	*…*	*…*
‘C’ ‘C’ ‘C’	*e*_*343,1*_	*e*_*343,2*_	*…*	*e*_*343,n-2*_

### Representation within heterogeneous structure information perspective

Recently graph-based deep learning methods have achieved great success in capturing topological information about biological entities [59]. As mentioned earlier, sufficiently utilizing the heterogeneous information of complex associations among drugs, proteins and diseases is a key point of DTIs identification and drug repositioning. Due to the different types of nodes and edges, meta-path-based topological patterns are used for systematic analyses of heterogeneous networks. Meta-path can be understood as the consecutive nodes and edges between two focused nodes, which can convert network structure to semantic sequence [60].

Specifically, the protein–drug-disease three-layer heterogeneous network can be regarded as a bidirected information graph $G = (V,E)$, where *V* indicates the set of entity nodes $v \in V$, and *E* denotes the set of association edges $e \in E$. Let *T*_*v*_ represents the set of entity types and *T*_*e*_ represents the set of association types. The schema of $S_{G} = (T_{v} ,T_{e} )$ describes the meta-path-structure of the heterogeneous graph *G*. The meta-path *M* is based on the schema *S*_*G*_, which can be represented as the form of $T_{{v_{1} }} \to ^{{T_{{e_{1} }} }} T_{{v_{2} }} \to ^{{T_{{e_{2} }} }} \mathop {......}\limits^{{}} \to ^{{T_{{e_{n - 1} }} }} T_{{v_{n} }}$. Meta-path essentially describes different association combinations of nodes, and different schema of meta-path have different semantics. Given a meta-path $m = (v_{1} ,v_{2} ,...v_{n} )$, which is based on the schema $S_{{G_{m} }}$, the types of all nodes have to belong to set *T*_*v*_, and the types of each $e_{i} = < v_{i} ,v_{i + 1} >$ in meta-path *m* must be the same as the corresponding $T_{{e_{i} }}$ in the schema of $S_{{G_{m} }}$. To generate meta-path instances, the node transition probability on step *i* can be defined as follows:

5 $$ P(v_{i + 1} |v_{i} ) = \left\{ \begin{gathered} \frac{1}{{|N_{{S_{{G_{m} }} }} (v_{i} )|}},{\text{ if }} < v_{i + 1} ,v_{i} > \in E,S_{{G_{m} }} (v_{i + 1} {) = }T_{{v_{i + 1} }} \hfill \\ 0,{\text{ else}} \hfill \\ \end{gathered} \right. $$

where the $N_{{S_{{G_{m} }} }} (v_{i} )$ indicates the neighbor nodes of *v*_*i*_ under the schema of $S_{{G_{m} }}$. In this work, due to each meta-path, whose length is greater than 5, consisting of the meta-paths, whose lengths are short than or equal to 5, we defined six basic types of meta-paths, as follows:

- **t-dr-t**: target-drug-target. The meta-path denotes that the targets related to the same drug should be similar.
- **t-dr-di-dr-t**: target-drug-disease-drug-target. The two drugs in the meta-path are related to the same disease, thus the two drugs should be similar. Furthermore, the two targets related to similar drugs should also be similar.
- **dr-dr**: drug-drug. The edge between drugs indicates that the two drugs have the same pharmacological characterization. So, these drugs should be similar.
- **dr-t-dr**: drug-target-drug. The meta-path denotes that the drugs related to the same target should be similar.
- **dr-di-dr**: drug-disease-drug. The meta-path denotes that the drugs related to the same disease should be similar.
- **t-dr-dr-t**: target-drug-drug-target. The meta-path denotes that the targets related to similar drugs should be similar.

Then, under each schema, drug or protein nodes are randomly selected as the starting node to generate the semantic sequence, which consists of 64 basic meta-path instances linked head-to-tail. Various schemas can fully capture the complex structure of the heterogeneous network, and various semantic sequence instances in each schema can fully capture specific association information. To effectively extract the feature vector of the association information, all drug or protein nodes are respectively used as the initial node to ensure structural integrality, and then we randomly select the initial node 500 times to obtain various sequences, which can ensure the structural diversity. Finally, the meta-path embedding model of CBOW is utilized to generate the embedding representations with 64 dimensions of sequences of multiple schemes. In the process of the training model, we remove the DTIs in the test set to avoid the disclosure of information by the semantic sequences.

### Representation within similarity information perspective

In order to fully adopt the compensation of features of the multimodal mechanism, we further exploit similarity information. Specifically, SIMCOMP [45] is used to compute the chemical structure similarity of drugs, which is based on common substructures between each pair of drugs. The similarity matrix of drug *S*_*d*_, which represents chemical space, can be obtained by the formula:

6 $$ S_{d} (i,j) = \frac{{|d_{i} \cap d_{j} |}}{{|d_{i} \cup d_{j} |}} $$

where *d*_*i*_ and *d*_*j*_ respectively indicate *i*-th and *j*-th drug. To calculate the sequence similarity of each pair of proteins to obtain the similarity matrix, which represents genomic space, the Smith-Waterman score is utilized, which is defined as follows:

7 $$ S_{g} (i,j) = \frac{{SW(p_{i} ,p_{j} )}}{{\sqrt {SW(p_{i} ,p_{i} )} \sqrt {SW(p_{j} ,p_{i} )} }} $$

where *p*_*i*_ and *p*_*j*_ respectively indicate *i*-th and *j*-th protein. Based on the previous work [46], we utilize the row of calculated similarity matrixes as the embedding vectors.

### Joint representation based on multiple information perspectives

After generating the final representation vector of each drug and protein under three single perspectives, multiple features should be merged effectively to obtain better generalization. The method of fusing them plays a crucial role during the training process. Most of the existing models simply concatenated the representation of drugs and proteins, and then input them into machine learning classifiers or DNN, which cannot deal with different types of noise and represent features in a meaningful way. Inspired by Baltrušaitis et al. [61] and Cao et al. [62], a joint representation framework based on the neural network is proposed to complete the multi-modal representation learning task.

**Fig. 2:**
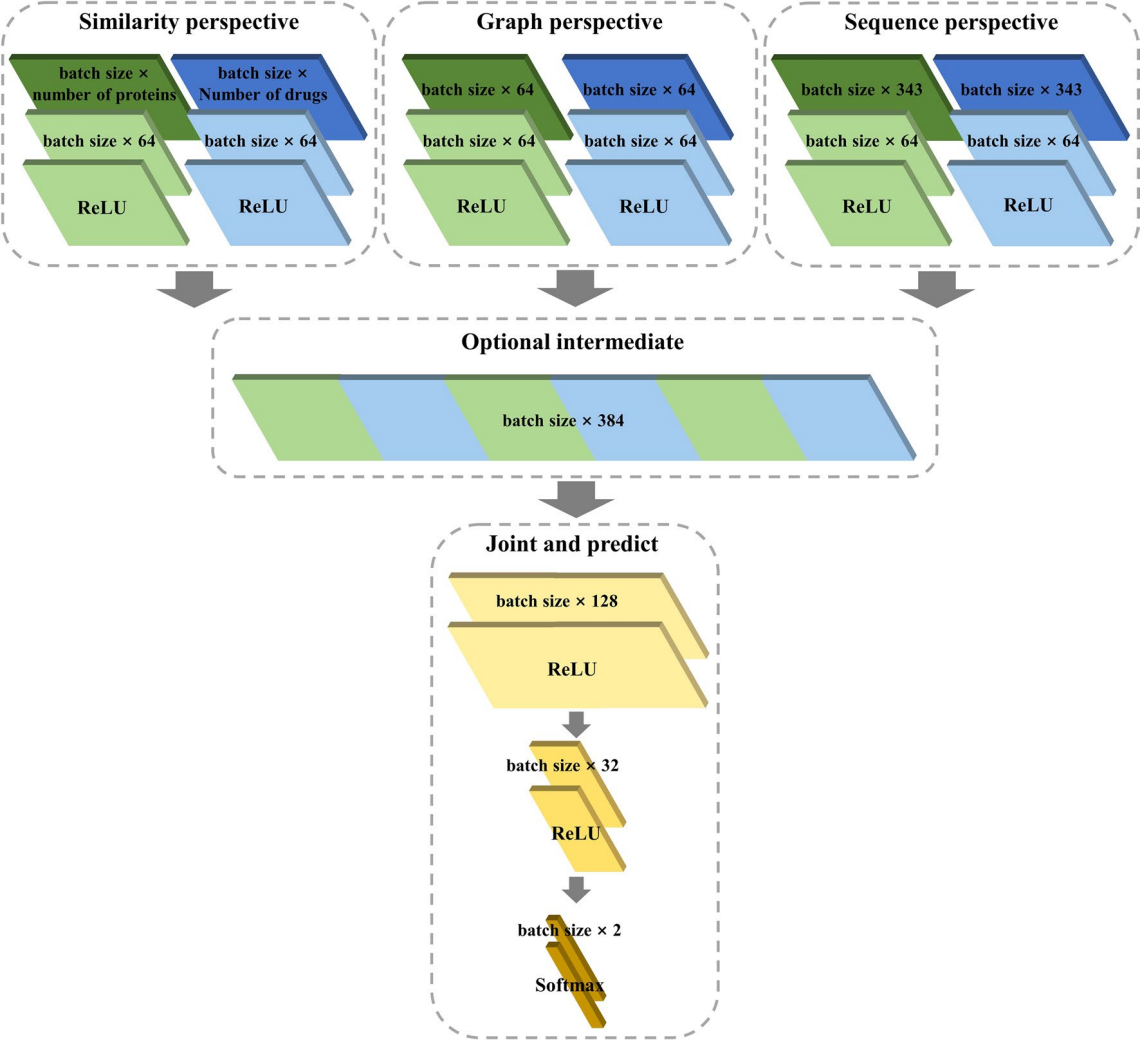
The detailed joint representation process and the parameter setting

The framework utilizes Y-shaped architecture, commonly used for DL-based predictive models [63]. As Fig. 2 shows, single modalities respectively begin with distinct individual layers, which have 64 neurons. Then all the modalities are projected into a joint space, which can be regarded as a common space, by the hidden layers [64]. The projection process of each representation vector *f*(*v*_*i*_) can be defined as the formula:

8 $$ h_{i} = {\text{ReLU}}(W_{M} f(v_{i} ) + b_{M} ) $$

where *W*_*M*_ and *b*_*M*_ respectively indicate the weight and bias of the hidden layer of modality *M* and the ${\text{ReLU}} = \max (0,x)$ is a function of linear rectification. Afterward, under the best projection from multiple spaces to the common space, each modality is aggregated into the joint multimodal representation through the function as follows:

9 $$ a_{i} = concat(h_{i}^{{d_{{M_{1} }} }} ,h_{i}^{{p_{{M_{1} }} }} ,h_{i}^{{d_{{M_{2} }} }} ,h_{i}^{{p_{{M_{2} }} }} ,h_{i}^{{d_{{M_{3} }} }} ,h_{i}^{{p_{{M_{3} }} }} ) $$

where $h_{i}^{{d_{{M_{j} }} }}$ and $h_{i}^{{p_{{M_{j} }} }}$ respectively are the representation vectors of *i*-th drug and protein in the joint space of *M*-th modality. Finally, the joint multimodal representation vector *a*_*i*_ is passed through two hidden layers, which respectively contain 128 neurons and 32 neurons, to obtain the identified results.

## Experimental results and discussion

### Evaluation criteria and experimental settings

In this work, to evaluate the performance of DeepMPF, five-fold cross-validation is applied. We construct the training set according to the procedure mentioned above. Additionally, six criteria are chosen to make the comprehensive evaluation of the robustness of the proposed model: the area under the ROC curves (AUC), accuracy (Acc.), sensitivity (Sen.), precision (Prec.), F1-score and Matthews’s Correlation Coefficient (MCC). The Acc., Sen., Prec., F1-score and MCC can be defined as the function:

10 $$ Acc. = \frac{TP + TN}{{TN + TP + FN + FP}} $$

11 $$ Prec. = \frac{TP}{{TP + FP}} $$

12 $$ Sen. = \frac{TP}{{TP + FN}} $$

13 $$ F1 - score = \frac{2 \times Prec. \times Sen.}{{Prec. + Sen.}} $$

14 $$ MCC = \frac{TP \times TN - FP \times FN}{{\sqrt {(TP + FP) \times (TN + FN) \times (TN + FP) \times (TP + FN)} }} $$

The mean value of each evaluation criterion can ensure a low-variance and unbiased evaluation. Besides, the binary-cross-entropy loss is employed to judge the proximity between the expected and the actual output. The adam optimizer is used and the dropout is set to 0.3 to reach the best performance. Due to the large difference in the size of all datasets, we respectively set the training batch of Enzyme dataset, GPCR dataset, Ion channel dataset and Nuclear receptor dataset as 128, 4, 16 and 2.

### Assessment of predictive performance

In the experiment, to evaluate the performance of DeepMPF, we apply the five-fold cross-validation method to four commonly used gold standard datasets. The positive and negative sample sets of drugs and proteins are meanly divided into five subsets according to the five-fold cross-validation. Then we randomly select one subset as the testing set, the seven-eighth remaining subsets are seen as the training set and the one-eighth remaining subsets are seen as the validation set. Finally, we plot the graphs and tables to analyze and summarize the experimental results.

**Fig. 3:**
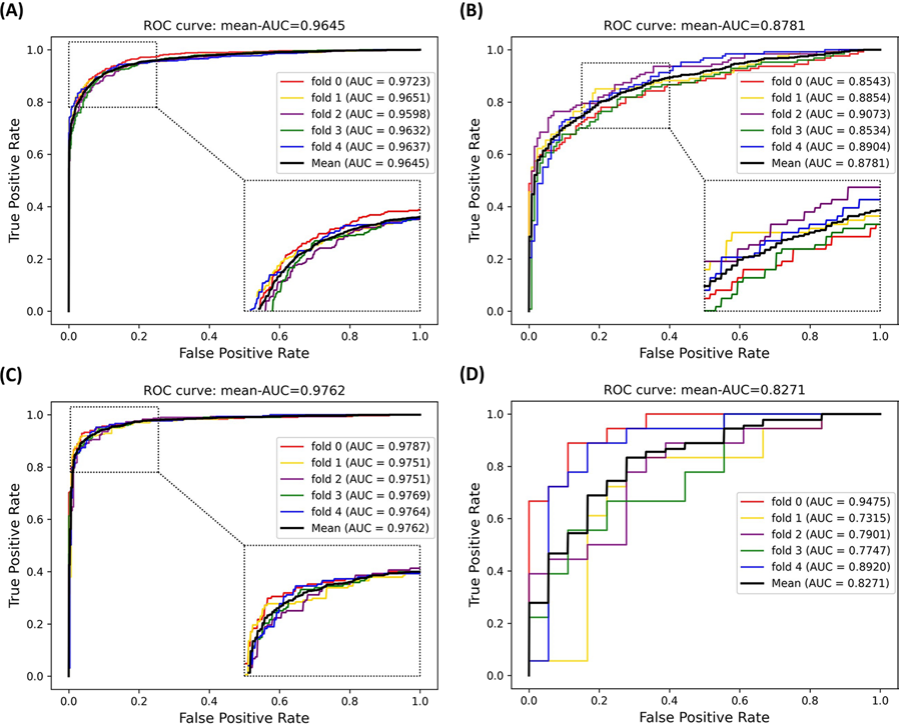
The ROC curves performed by DeepMPF framework based on the four gold standard datasets. **A**, **B**, **C** and **D** respectively indicate the results of Enzyme, GPCR, Ion channel and Nuclear receptor

As Table 3 shown, the average scores of ACC reach 0.9057, 0.7960, 9305 and 0.7500 in all gold standard datasets. Besides, our framework respectively achieves average AUC of 0.9645, 0.8781, 0.9762 and 0.8271, which is shown in Fig. 3. After analyzing the results, on the datasets of Enzyme and Ion channel, our method achieves a better performance of high values of Acc. and AUC and slight fluctuation of results. While, on the datasets of Nuclear receptor, relatively poor results are obtained. The difference in performance is mainly caused by the size of datasets, that too small size of datasets can limit the capability of DTIs prediction. And another reason possibly is that DeepMPF is more sensitive to the protein type of Enzyme and Ion channel. However, we still reach the high AUC of 0.8832 on the smallest dataset, which reflects the proposed framework based on multi-modal representation learning is suitable for identifying unknown DTIs.

**Table 3:** Five-Fold cross-validation results on four gold-standard datasets through DeepMPF

Dataset	Fold	Acc	Prec	Sen	F1	MCC
Enzyme	1	0.9111	0.9242	0.8957	0.9097	0.8226
	2	0.9111	0.9397	0.8786	0.9081	0.8240
	3	0.8973	0.9218	0.8682	0.8942	0.7959
	4	0.9034	0.9083	0.8974	0.9028	0.8069
	5	0.9058	0.9217	0.8870	0.9040	0.8122
	Average	0.9057 ± 0.0058	0.9231 ± 0.0112	0.8854 ± 0.0122	0.9038 ± 0.0061	0.8123 ± 0.0116
GPCR	1	0.7756	0.8571	0.6614	0.7466	0.5661
	2	0.8031	0.8667	0.7165	0.7845	0.6156
	3	0.8228	0.8596	0.7717	0.8133	0.6491
	4	0.7756	0.8365	0.6850	0.7532	0.5604
	5	0.8031	0.8291	0.7638	0.7951	0.6082
	Average	0.7960 ± 0.0203	0.8498 ± 0.0161	0.7197 ± 0.0481	0.7785 ± 0.0282	0.5999 ± 0.0369
Ion channel	1	0.9375	0.9360	0.9392	0.9376	0.8750
	2	0.9407	0.9610	0.9186	0.9393	0.8822
	3	0.9169	0.9271	0.9051	0.9160	0.8341
	4	0.9254	0.9435	0.9051	0.9239	0.8516
	5	0.9322	0.9264	0.9390	0.9327	0.8645
	Average	0.9305 ± 0.0096	0.9388 ± 0.0143	0.9214 ± 0.0171	0.9299 ± 0.0098	0.8615 ± 0.0192
Nuclear receptor	1	0.8333	0.9286	0.7222	0.8125	0.6838
	2	0.7222	0.7857	0.6111	0.6875	0.4558
	3	0.6667	0.7500	0.5000	0.6000	0.3536
	4	0.6944	0.7059	0.6667	0.6857	0.3895
	5	0.8333	0.8750	0.7778	0.8235	0.6708
	Average	0.7500 ± 0.0786	0.8090 ± 0.0913	0.6556 ± 0.1069	0.7219 ± 0.0947	0.5107 ± 0.1565

**Fig. 4:**
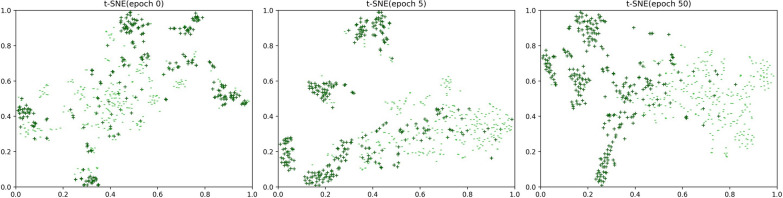
Visualization in the 2D space by t-SNE of the learned DTI embeddings on the dataset of Ion channel

With the exception of the quantitative analysis, we visualize the results of DTIs identification on the testing set of Ion channel by t-SNE [65] to further demonstrate the superior predictive ability of our framework. As shown in Fig. 4, the ‘ + ’ with the color of dark green indicates the positive sample, and ‘-’ with the color of lime green indicates the negative sample. With the number of training epoch enhancing, two types of samples can be gradually separated, and finally can be basically identified.

### Ablation experiments

To better test the contribution levels of the different single modalities of DeepMPF in DTIs identification, the in-depth ablation study has been conducted with extensive experiments. To this end, we denote our framework as the complete multimodal model and perform the leave-one-out validation on each modality part of the model to test the single modality with the largest effect. Additionally, we also test the framework with only sequence modal information, which can partly reflect the overall performance of the traditional method based on similarity. ‘**Without sequence**’ represents our framework without the modality information of the sequences of drugs and proteins. ‘**Without meta-path**’ denotes our framework without the modality information of the heterogeneous structure association among drugs, proteins and diseases. ‘**Without similarity**’ means our framework without the modality information of the similarity of drugs and proteins. ‘**Sequence only**’ indicates our framework only using the modality information of the sequences of drugs and proteins.

We draw Table 4 to make analyzation. At first, the value of AUC is decline in each ablation experiment, and obviously, the modality information of the heterogeneous structure performs the most significant contribution to the first three datasets. However, in the last dataset, this modality provides little contribution compared with other modalities. One of the possible reasons is the large heterogeneous network may bring too much noise information for small datasets, which makes the representation pattern of sequence and similarity more useful and more suitable. In the last ablation experiment, only the result on the last dataset is anomalous, whose value of AUC is higher than the AUC values of the first and third ablation experiments but lower than the AUC values of the second ablation experiments. It also indicates that the modality information of the sequences is more suitable for the small dataset. Even so, the multi-modal model can achieve the optimum performance only when fully exploiting and fusing all the single modalities. Undeniably, the modality information of the heterogeneous structure plays a crucial role in our framework, thus related impact factors are discussed in the later sections.

**Table 4:** Results of ablation test on DeepMPF for AUC

Dataset	DeepMPF (ours)	Without sequence modality (-△)	Without heterogeneous structure modality (-△)	Without similarity modality (-△)	Only modality of sequence (-△)
Enzyme	0.9645	0.9618 (− 0.0027)	0.9405 (**− 0.0240**)	0.9503 (− 0.0142)	0.8462 (− 0.1183)
GPCR	0.8781	0.8628 (− 0.0153)	0.8403 (**− 0.0378**)	0.8573 (− 0.0208)	0.7599 (− 0.1182)
Ion channel	0.9762	0.9747 (− 0.0015)	0.9463 (**− 0.0299**)	0.9657 (− 0.0105)	0.7341 (− 0.2421)
Nuclear receptor	0.8271	0.7870 (**− 0.0401**)	0.8131 (− 0.0140)	0.7938 (− 0.0333)	0.8050 (− 0.0221)

The bold values represent the maximum drop value of AUC on each dataset among the comparisons except the term of sequence only

### Influence of embedding dimension of the heterogeneous graph

**Fig. 5:**
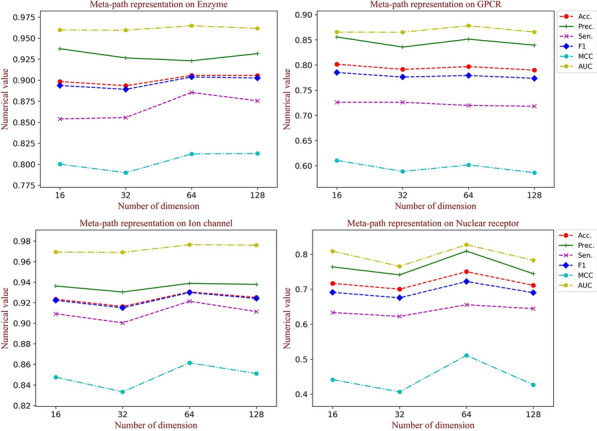
The performance with different embedding dimensions of the heterogeneous graph on each dataset

As the discussion in the previous section, modality information of the heterogeneous graph structure crucially affects the identification results. Thus, we comprehensively evaluate predictive performance to analyze the influence of different dimensions, containing 16, 32, 64 and 128. As shown in Fig. 5, with the number of latent factors increasing, the performance roughly presents a trend of the first rise and then decline with a small magnitude. In our study, 64 is chosen to obtain the rich topology information, which can capture adequate information without much noise introduction. Finally, it can be demonstrated that DeepMPF has a stable capability of DTIs prediction over a wide range of embedding representation dimensions.

### Influence of learning strategies of heterogeneous graph

**Fig. 6:**
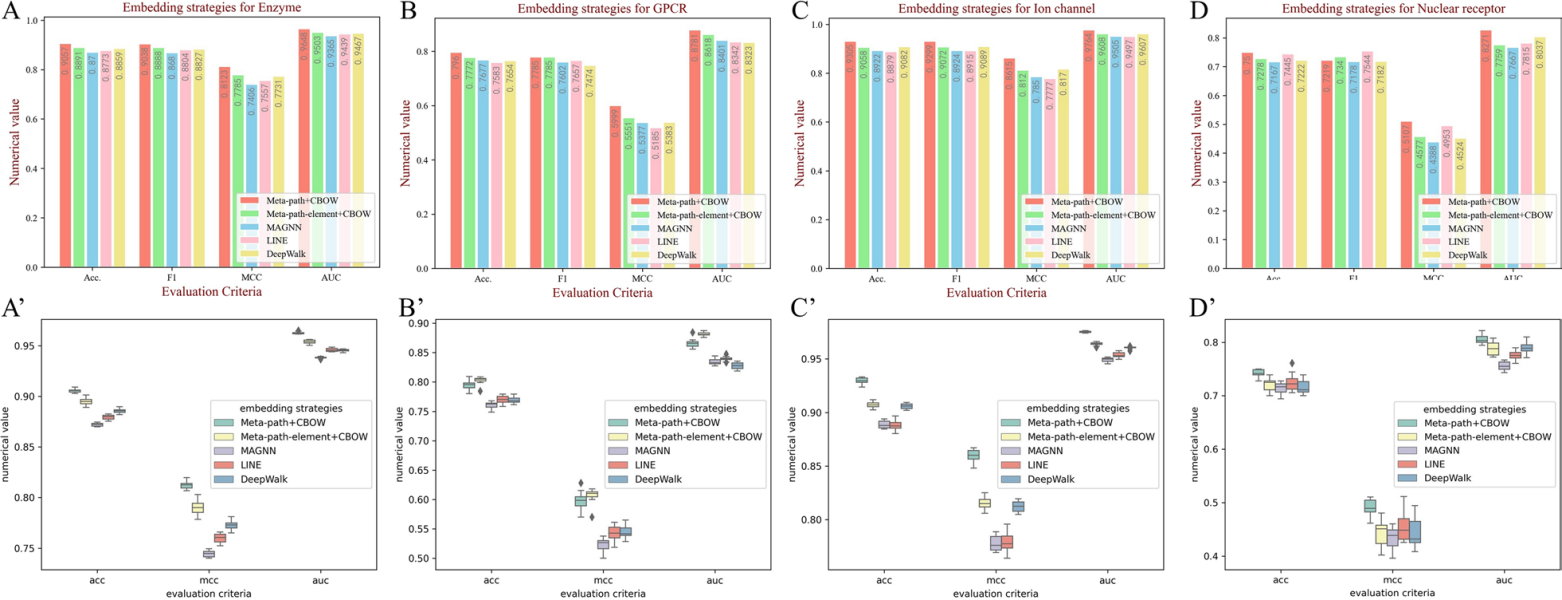
The performance of different learning strategies of the heterogeneous graph on each dataset

In our framework, we propose six schemas based on meta-path to fully capture the topological structure of the heterogeneous graph, and then, the latent semantic information is extracted by CBOW. To verify the validity of our learning strategies on the heterogeneous graph, we test and analyze the other four learning strategies. First, based on our meta-path schemas, we directly regard each basic meta-path instance as a semantic sequence, which can pay more attention to the local heterogeneous structure. Second, we used MAGNN, proposed by Fu et al. [66], which is also a heterogeneous graph embedding method. Additionally, two graph embedding methods of LINE [67] and DeepWalk [68] are used for comparison. For a fair, the embedding dimension is the same and the parameters of each embedding method are default. As Fig. 6A–D shows, although there are a few fluctuations in some evaluation criteria, our learning strategy can reach the best performance, which is attributed to our method can preserve the high-order nonlinear structure and catch latent information of the deep heterogeneous graph. To verify the results of the comparison experiments are truly significant, we utilized the statistical learning method to plot boxplots, as shown in Fig. 6A’–D’.

### Comparison with different classification methods

**Fig. 7:**
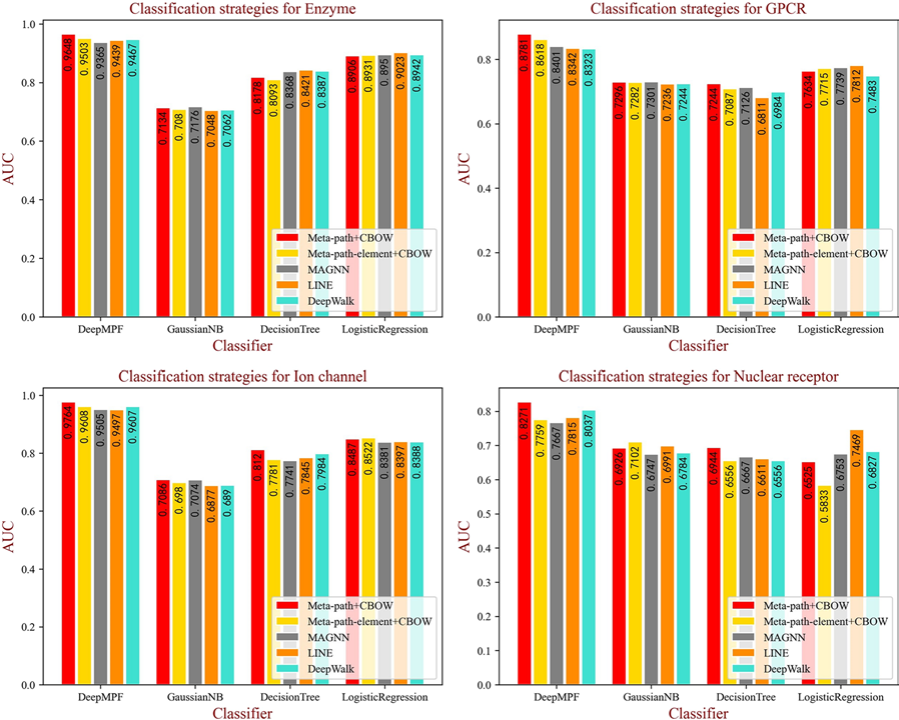
The AUC values of different classifiers with various embedding strategies on each dataset

In our DeepMPF framework, we fully exploit multi-perspective features to identify unknown DTIs through multi-modal joint representation with Y-shape structure neural network. To test the effectiveness of our framework, three classifiers are used to compare the AUC value, regarded as an important criterion for binary classification, with various learning strategies of the heterogeneous graph mentioned above on the four datasets. Specifically, all the feature descriptors are unchanged to ensure a fair comparison. The results of prediction through Gaussian NB, Decision Tree and Logistic Regression are shown in form of histograms for intuitive comparison in Fig. 7. Obviously, although our embedding strategies did not obtain the best performance with the other three classifiers, our embedding strategy achieved a promising performance with our joint representation learning framework. It is worth noting that, when using our framework, the lowest value of AUC is still higher than the other comparison methods, which further demonstrates our framework can advance the representation of multi-perspective.

### Comparison with other state-of-the-art methods

Plenty of computational methods have been proposed for DTI prediction [86]. To more objectively verify the effectiveness and stability of DeepMPF, we compared the predictive performance through the value of AUC, which is frequently used to measure the performance of the model, with other 13 state-of-the-art computational models in the same four datasets based on fivefold cross-validation. The comparison method can be partitioned into two classes: Lower-view and Higher-view [84]. Table 5 described the results of AUC and the results of other performance metrics are reported in Additional file 1: Table S1. It can be seen that compared with other methods, our method respectively obtains the highest AUC value on the datasets of Enzyme and Ion channel with outstanding improvements of 0.0106 ~ 0.1225 and 0.0076 ~ 0.0862. However, on the dataset of Nuclear receptor and GPCR, the AUC value of our model is respectively lower than the method of Li et al. and SAR. A possible reason is that the most critical modality of heterogeneous structure in our framework is more suitable for a large network. Thus, applying DeepMPF to the datasets of Nuclear receptor and GPCR just obtains the general predictive results. Notably, the method of Li et al. and SAR only get the highest AUC value on the corresponding dataset, but relatively low AUC values on the other datasets, which indicates these methods have poor generalization. Additionally, the method of lNeuRank performs the second-highest AUC values on both Enzyme and Nuclear receptor datasets and acceptable performance on the other two datasets, which means it has high generalization ability. And then, our method still shows better performance than lNeuRank. On the whole, although the performance of DeepMPF framework outperforms many other state-of-the-art methods, there is still improvement room for our method.

**Table 5:** AUC values of comparing with state-of-the-art methods on gold-standard datasets

Model view	Method	Enzyme	GPCR	Ion channel	Nuclear receptor
Lower-view	Zhan et al*.* [69]	0.9532	0.8882	0.9349	0.8199
	Li et al. [70]	0.9288	0.8856	0.9171	**0.9300**
	Pan et al. [30]	0.9498	0.8775	0.9270	0.7755
	SAR [73]	0.9486	**0.8902**	0.9428	0.8822
	MLCLE [74]	0.8420	0.8500	0.7950	0.7900
	RFDT [75]	0.9150	0.8450	0.8900	0.7230
	DeepDTIs [31]	0.9067	0.8603	0.9417	0.8043
Higher-view	DASPfind [26]	0.9291	0.8810	0.9068	0.8527
	DT‑Hybrid [71]	0.8980	0.8387	0.9200	0.6995
	NRWRH [72]	0.9289	0.8493	0.9156	0.7390
	CMF [76]	0.8785	0.8244	0.8974	0.7637
	BRDTI [77]	0.8834	0.8487	0.9234	0.7962
	lNeuRank [78]	0.9539	0.8615	0.9686	0.7832
	DeepMPF (our)	**0.9645 ± 0.0046**	0.8782** ± **0.0236	**0.9762 ± 0.0015**	0.8272** ± **0.0894

The bold values represent the higher values in each dataset

### Application in drug repositioning

As the above description, predicting potential DTIs can provide great help for the task of drug repositioning. The outbreak of COVID-19 has caused millions of deaths since 2019, thus, it is crucial to repurpose old drugs for new therapeutic [79]. To apply our method in real life and to validate that DeepMPF can help drug repositioning, we used DeepMPF to find therapeutic drugs for COVID-19-related proteins. In this study, three homo sapiens proteins, inextricably related to COVID-19 are utilized to conduct the drug repositioning task. The related proteins are shown in Table 6.

**Table 6:** Three homo sapiens proteins related to COVID-19

Protein name	UniProtKB ID	Description	Evidence
Apolipoprotein E	P02649	Allele APOE*4 is strongly related to COVID-19	PMID: 33450186
Angiotensin-converting enzyme 2	Q9BYF1	It can increase the affinity for SARS-CoV-2 spike protein	PMID: 32753553
Elongation factor 1-alpha 1	P68104	It is required for viral replication and translation of viral proteins	PMID: 33495306

The dataset of proDB is utilized to train the predictive model after deleting the DTIs of three testing proteins and DDAs of COVID-19 from the train set, which can avoid label leakage. Specifically, the tested proteins are respectively paired with each drug. Then, the multi-modal information of pairwise DTI is fed into the predictive model. Finally, the score of each DTI pair can be generated. We comprehensively ranked the drug scores representing the probability associated with COVID-19, and then respectively selected the top 5 drugs in ascending order, which is reported in Table 7. We note that 5, 3 and 5 out of the top 5 drugs identified have been validated by the related publications. According to the evaluation of the European Food Safety Authority (EFSA), minerals of zinc and copper, etc. play a crucial role in the immune system, which can reduce the harm of COVID-19 [80]. As the first part of Table 7 shows, copper, zinc and zinc salt exhibit the highest scores. Copper can be found in several supplements and vitamins and is critical to the function of many enzymes, like cytochrome c oxidase. The detailed process of drug function can be found in the evidence publications, and the detailed scores are reported in Additional file 2: Table S2–S4.

**Table 7:** The predicted top 10 drugs associated with COVID-19 based on three related proteins

Related protein	Drug name	DrugBank ID	Score	Evidence
Apolipoprotein E (P02649)	Copper	DB09130	0.9953	PMID: 32503814
	Zinc chloride	DB14533	0.9936	PMID: 34972736
	Silver	DB12965	0.9912	PMID: 32958250
	Zinc acetate	DB14487	0.9884	PMID: 32522597
	Zinc	DB01593	0.9883	PMID: 32319538; PMID: 33094446
Angiotensin-converting enzyme 2 (Q9BYF1)	Cefoxitin	DB01331	0.9894	N.A
	Cloxacillin	DB01147	0.9890	PMID: 35378738
	Piperacillin	DB00319	0.9869	PMID: 33576584
	Moexipril	DB00691	0.9860	PMID: 34631362; PMID: 34458381
	Cefmetazole	DB00274	0.9836	N.A
Elongation factor 1-alpha 1 (P68104)	Copper	DB09130	0.9711	PMID: 32503814
	Zinc chloride	DB14533	0.9558	PMID: 34972736
	NADH	DB00157	0.9391	PMID: 33132205
	Caffeine	DB00201	0.8998	PMID: 34067243; PMID: 33193427
	Flavin adenine dinucleotide	DB03147	0.8701	PMID: 32294562; PMID: 34823857

Additionally, to further explain the reliability of DeepMPF, we also apply our framework to predict potential therapeutic drugs for HIV-related protein and then select the top 20 drugs in ascending probability order to analyze. During the course of HIV treatment, the patients often take at least three drugs to suppress viral replication. However, the competition for Cytochrome P450 can reduce efficacy in HIV treatment. Accurate identification of DTIs can effectively avoid a decrease in drug efficacy. In this experiment, the protein of CYP3A4, closely related to HIV, is chosen as the test target. The results are shown in Table 8.

**Table 8:** The top 20 drugs interacting with CYP3A4

Rank	Drug name	DrugBank ID	Evidence	Rank	Drug name	DrugBank ID	Evidence
1	Amitriptyline	DB00321	Confirmed	11	Ketamine	DB01221	Confirmed
2	Haloperidol	DB00502	Confirmed	12	Enflurane	DB00228	N.A
3	Brigatinib	DB12267	Confirmed	13	Melatonin	DB01065	N.A
4	Aripiprazole	DB01238	Confirmed	14	Nandrolone decanoate	DB08804	N.A
5	Methadone	DB00333	Confirmed	15	Glycyrrhizic acid	DB13751	N.A
6	Pyrimethamine	DB00205	N.A	16	Dabrafenib	DB08912	Confirmed
7	Rhein	DB13174	Confirmed	17	Naringenin	DB03467	N.A
8	Olaparib	DB09074	Confirmed	18	Tenofovir disoproxil	DB00300	N.A
9	Ponatinib	DB08901	Confirmed	19	Tepotinib	DB15133	Confirmed
10	Zanubrutinib	DB15035	Confirmed	20	Nilotinib	DB04868	Confirmed

**Fig. 8:**
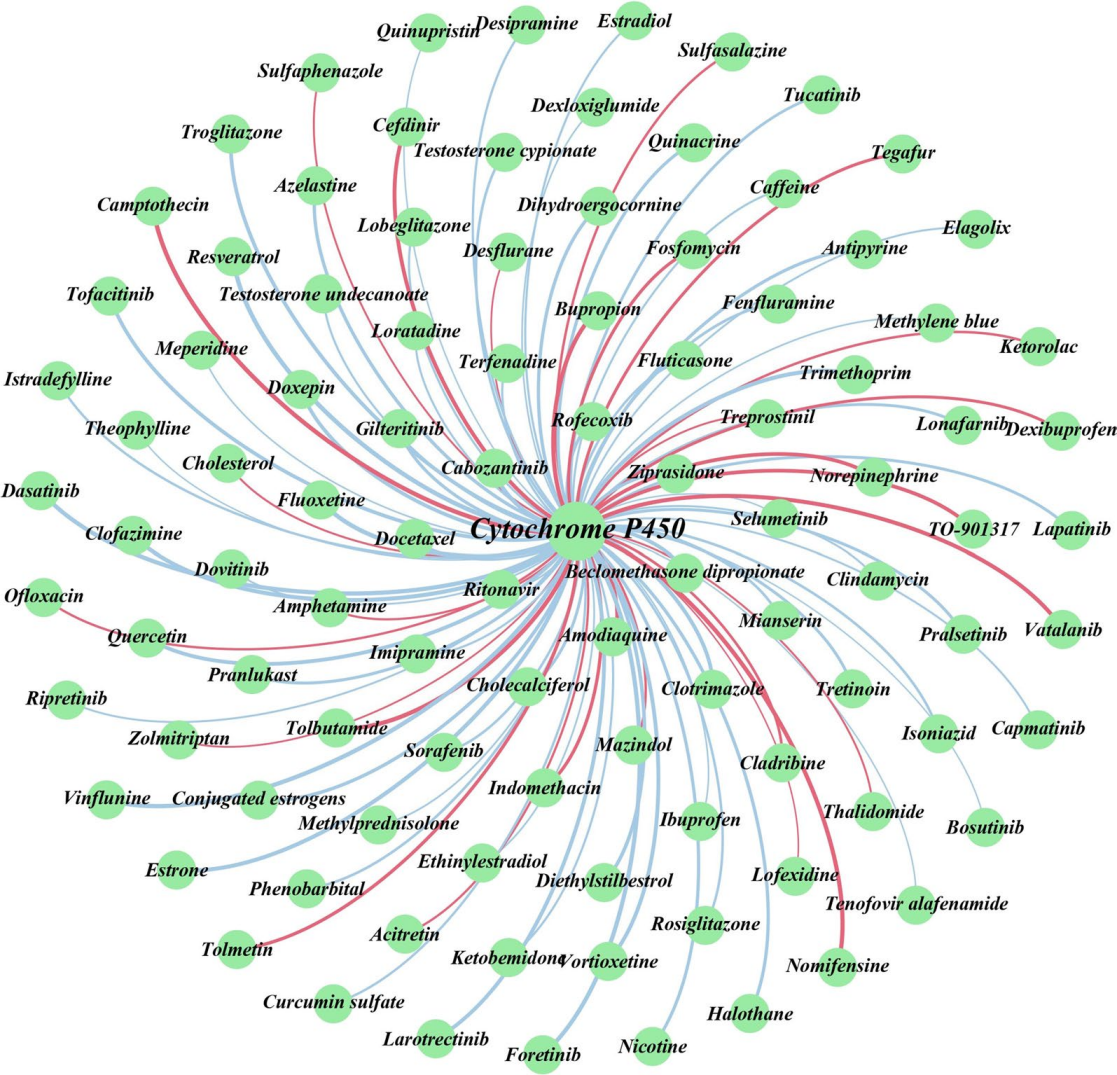
Network visualization of known and newly discovered DTIs of the top 100 HIV-related proteins. Red lines mean the interactions with no support and blue lines mean confirmed interactions. Thick and thin lines represent the possibility of potential interactions

From the table, it can be found that 9 of the top 10 drugs interacting with CYP3A4 are confirmed by DrugBank, and all of the top five drugs are confirmed, which demonstrates our method has a good capacity to identify unknown DTIs. To clearly and completely observe the results of identifying DTIs, the novelly identified and known interactions of the top 100 are visualized in Fig. 8. The complete prediction scores are reported in Additional file 2: Table S1. It can be seen that 72 of 100 interactions are identified successfully by DeepMPF. More importantly, the remaining unconfirmed 28 interactions are given high confidence, especially pyrimethamine, to deserve further study. In summary, DeepMPF has promising performance in discovering potential DTIs above analysis. Moreover, our work provides some DTIs with high confidence, which can facilitate the progress of drug repositioning through further wet-lab assays.

### Molecular docking experiment

To further prove the credibility of DeepPMF, molecular docking experiments [81] are conducted on the top 14 drugs listed in Table 8. The intermolecular binding ability of each drug with CYP3A4 is computed. Specifically, the structure of CYP3A4 (PDB ID: 1W0E) is downloaded from RCSB PDB [82], and the structures of the drug are collected from PubChem [17]. Then we utilized AutoDockTools [83] to process the structure files, and put the processed files into AutoDock software to complete the molecular docking experiment of protein and ligands. The binding energies, i.e., binding free energy, of molecular docking are shown in Table 9. The lower binding energy indicates the stronger binding of the molecular.

**Table 9:** The binding energies between predicted drugs and the protein of CYP3A4

Drug name	Binding energy (kcal/mol)	Drug name	Binding energy (kcal/mol)
Amitriptyline	− 4.93	Olaparib	− 4.14
Haloperidol	− 3.28	Ponatinib	− 4.19
Brigatinib	− 3.46	Zanubrutinib	− 4.08
Aripiprazole	− 3.62	Ketamine	− 3.99
Methadone	− 3.77	Enflurane	− 1.91
Pyrimethamine	− 4.45	Melatonin	− 3.83
Rhein	− 3.97	Nandrolone decanoate	− 4.89

**Fig. 9:**
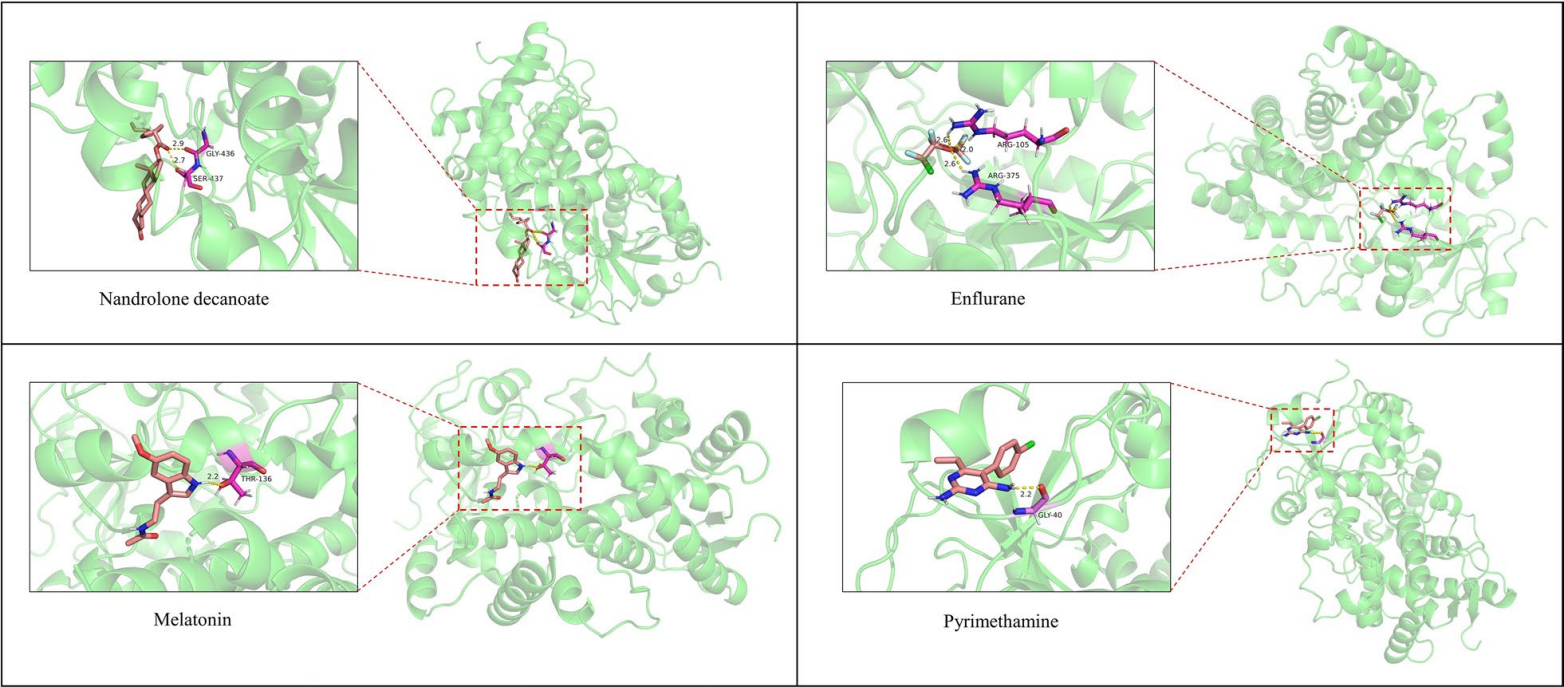
Molecular docking results for Nandrolone decanoate, Enflurane, Melatonin and Pyrimethamine bound with HIV-related protein CYP3A4

We note that for the top 5 confirmed drugs Amitriptyline, Haloperidol, Brigatinib, Aripiprazole and Methadone, their binding energy with CYP3A4 respectively are − 4.93 kcal/mol, − 3.28 kcal/mol, − 3.46 kcal/mol, − 3.62 kcal/mol and − 3.77 kcal/mol. The binding energies of the unconfirmed drugs of Pyrimethamine, Enflurane, Melatonin and Nandrolone decanoate are also positioned at a relatively lower level, even lower than several confirmed drugs. Moreover, their binding sites are presented in Fig. 9. Overall, these analyses further demonstrate the interactions between the four drugs and CYP3A4 are possibly existed, however, the molecular docking experiment just provides an interaction possibility, and the more accurate results entail in-depth wet-lab experiments to verify.

## Conclusion

In this study, we proposed a framework of DeepMPF to predict candidate DTIs through learning multi-modal information. Related data is collected to construct a protein–drug-disease association heterogeneous network benefitting extracting the deep network structure information. To comprehensively capture complex topology structures crossing the chemical and biological space, we design six meta-path schemas used to learn network heterogeneity semantics information preserving the high-order nonlinear structure and extracting latent information. The sequence feature and similarity feature are fully utilized to ensure complementing information. The joint representation learning module is designed to effectively fuse different modality information as highly representative comprehensive feature descriptors and calculate the probability of interaction. Through comparison with state-of-the-art methods and analysis of classification or feature extraction strategies, it can be concluded our method achieved better performance and have the reliable ability to predict DTI. Additionally, to verify the efficacy of adopting DeepMPF in real-life problems, the experiment of drug repositioning on COVID-19 and HIV and the further analysis of molecular docking experiments demonstrate our method also has a great role in drug discovery. Furthermore, an online prescreening platform is built for related researchers and biologists to validate possible interactions from the perspective of chemogenomic and biomedicine. The prescreening platform is freely available at http://120.77.11.78/DeepMPF/. Code is available at https://github.com/MrPhil/DeepMPF. In conclusion, the experimental results demonstrate that DeepMPF is a reliable prescreening tool for further study and validate the mechanism of the DTIs. In the future, we will further improve the performance and generalization of the model by incorporating more information and using self-attention to enhance the drug development process.

## Supplementary Information

**Additional file 1: Table 1. **Results of comparing with state-of-the-art methods on gold-standard datasets.

**Additional file 2.** The complete prediction scores.

## Data Availability

DeepMPF is also publicly available as an online predictor at: http://120.77.11.78/DeepMPG/. The datasets used and/or analysed during the current study are available from the corresponding author upon reasonable request.
