# Supplementary material for: DeepMPF: deep learning framework for predicting drug–target interactions based on multi-modal representation with meta-path semantic analysis
Source: J Transl Med. 2023 Jan 25;21:48. doi: 10.1186/s12967-023-03876-3 (PMC9876420; doi:10.1186/s12967-023-03876-3)
Supplement: Supplementary file 1 — Additional file 1: Table 1. Results of comparing with state-of-the-art methods on gold-standard datasets. [file 12967_2023_3876_MOESM1_ESM.docx]

Table 1. Results of comparing with state-of-the-art methods on gold-standard datasets

| Dataset | Method | Acc. | Prec. | Sen. | AUC |
| --- | --- | --- | --- | --- | --- |
| Enzyme | Zhan *et al*. | 0.8909 | 0.9033 | 0.8756 | 0.9532 |
|  | Li *et al*. | 0.9316 | 0.9318 | 0.9290 | 0.9288 |
|  | Pan *et al*. | 0.8921 | 0.9077 | 0.8730 | 0.9498 |
|  | SAR | 0.9031 | (−) | 0.9010 | 0.9486 |
|  | MLCLE | (−) | (−) | (−) | 0.8420 |
|  | RFDT | 0.9130 | 0.9290 | 0.8940 | 0.9150 |
|  | **DeepMPF (our)** | **0.9057±0.0058** | **0.9231±0.0112** | **0.8854±0.0122** | **0.9645±0.0046** |
| GPCR | Zhan *et al*. | 0.8205 | 0.8148 | 0.8307 | 0.8882 |
|  | Li *et al*. | 0.8937 | 0.8940 | 0.8927 | 0.8856 |
|  | Pan *et al*. | 0.8102 | 0.8151 | 0.8038 | 0.8775 |
|  | SAR | 0.8468 | (−) | 0.8254 | 0.8902 |
|  | MLCLE | (−) | (−) | (−) | 0.8500 |
|  | RFDT | 0.8410 | 0.8410 | 0.8400 | 0.8450 |
|  | **DeepMPF (our)** | **0.7960±0.0203** | **0.8498±0.0161** | **0.7197±0.0481** | **0.8782±0.0236** |
| Ion channel | Zhan *et al*. | 0.8753 | 0.8728 | 0.8776 | 0.9349 |
|  | Li *et al*. | 0.9173 | 0.9090 | 0.9265 | 0.9171 |
|  | Pan *et al*. | 0.8549 | 0.8558 | 0.8540 | 0.9270 |
|  | SAR | 0.8891 | (−) | 0.8938 | 0.9428 |
|  | MLCLE | (−) | (−) | (−) | 0.7950 |
|  | RFDT | 0.8910 | 0.8760 | 0.9120 | 0.8900 |
|  | **DeepMPF (our)** | **0.9305±0.0096** | **0.9388±0.0143** | **0.9214±0.0171** | **0.9762±0.0015** |
| Nuclear receptor | Zhan *et al*. | 0.7333 | 0.7606 | 0.7153 | 0.8199 |
|  | Li *et al*. | 0.9222 | 0.8867 | 0.9662 | 0.9300 |
|  | Pan *et al*. | 0.7444 | 0.7231 | 0.7817 | 0.7755 |
|  | SAR | 0.8374 | (−) | 0.8235 | 0.8822 |
|  | MLCLE | (−) | (−) | (−) | 0.7900 |
|  | RFDT | 0.7110 | 0.6800 | 0.7590 | 0.7230 |
|  | **DeepMPF (our)** | **0.7500±0.0786** | **0.8090±0.0913** | **0.6556±0.1069** | **0.8272±0.0894** |

The symbol of (−) indicates the evaluation criteria are not reported in the original articles.
